# Analysis of Microtubule-Associated-Proteins during IBA-Mediated Adventitious Root Induction Reveals KATANIN Dependent and Independent Alterations of Expression Patterns

**DOI:** 10.1371/journal.pone.0143828

**Published:** 2015-12-02

**Authors:** Mohamad Abu-Abied, Inna Mordehaev, Gujulla B Sunil Kumar, Ron Ophir, Geoffrey O. Wasteneys, Einat Sadot

**Affiliations:** 1 The Institute of Plant Sciences, The Volcani Center, ARO, Bet-Dagan, Israel; 2 Department of Botany, The University of British Columbia, Vancouver, British Columbia, Canada; Iwate University, JAPAN

## Abstract

Adventitious roots (AR) are post embryonic lateral organs that differentiate from non-root tissues. The understanding of the molecular mechanism which underlies their differentiation is important because of their central role in vegetative plant propagation. Here it was studied how the expression of different microtubule (MT)-associated proteins (MAPs) is affected during AR induction, and whether expression differences are dependent on MT organization itself. To examine AR formation when MTs are disturbed we used two mutants in the MT severing protein KATANIN. It was found that rate and number of AR primordium formed following IBA induction for three days was reduced in *bot1-1* and *bot1-7* plants. The reduced capacity to form ARs in *bot1-1* was associated with altered expression of MAP-encoding genes along AR induction. While the expression of *MAP65-4*, *MAP65-3*, *AURORA1*, *AURORA2* and *TANGLED*, increased in wild-type but not in *bot1-1* plants, the expression of *MAP65-8* and *MDP25* decreased in wild type plants but not in the *bot1-1* plant after two days of IBA-treatment. The expression of *MOR1* was increased two days after AR induction in wild type and *bot1-1* plants. To examine its expression specifically in AR primordium, *MOR1* upstream regulatory sequence was isolated and cloned to regulate GFP. Expression of GFP was induced in the primary root tips and lateral roots, in the pericycle of the hypocotyls and in all stages of AR primordium formation. It is concluded that the expression of MAPs is regulated along AR induction and that reduction in *KATANIN* expression inhibits AR formation and indirectly influences the specific expression of some MAPs.

## Introduction

ARs are roots that differentiate from non-root tissues, as a response to endogenous or exogenous auxin signaling [1]. The tissues that give rise to AR founder cells are usually inner meristematic tissues such as the cambium in mature tissues [2, 3] or the pericycle in hypocotyls [4]. ARs are often compared to lateral roots (LRs), which are developed from the primary root [5] and like ARs, are post-embryonic lateral organs. Indeed ARs and LRs share similarities [6] but also differences [7].

In a recent study, the role of MTs in AR formation was investigated. It was found that subtle perturbations to MT dynamics can uncouple cell division from cell differentiation during AR induction [8]. It was shown that if AR were induced by IBA when MTs were partially disrupted by oryzalin or by transferring plants of the *mor1-1* temperature-sensitive mutant to a restrictive temperature, amorphous clusters of dividing cells were formed rather than AR primordium. In these clusters, which were as big as stage V primordium, MTs were mainly randomly oriented, cell wall properties were changed, PIN1 polarization and auxin maxima were impaired and the differentiation of the root epidermal cell layer was inhibited [8]. In the same study it was shown that while a mutant in *KATANIN*, *bot1-1* formed fewer ARs, a plant overexpressing RIC1 that activates KATANIN [9], formed more ARs compared to wild type plants. Curiously, it was reported that *bot1-7* as well as *ric1* mutant plants made more flowers than wild type plants when auxin transport was blocked [10] suggesting that these genes can have differential effects on post embryonic organogenesis. KATANIN is a MT severing protein that is involved with MT responses to ROP GTPase signaling [9], auxin signaling [11], blue light [12] and mechanical signaling [13]. The latter is highly relevant to AR formation because like LR, AR are differentiated from inner layers perceiving mechanical signaling from the upper layers above them. Studies on LRs have shown that primordium cells perform a mechanical signaling cross talk with the cell layers above them in different ways [14, 15], which eventually leads to the separation of the upper layers that enables LR emergence. In turn, it has been shown that inhibition of cell layer separation above the LR primordium by specifically suppressing auxin signaling in these layers or by suppressing *INFLORESCENCE DEFICIENT IN ABSCISSION* (*IDA*) abscission signaling affects LR primordium morphogenesis and differentiation [14, 16, 17]. These studies suggest that the pressure exerted by the upper layers needs to be gradually reduced during induction, to allow not only root emergence, but also proper LR differentiation. When primordium cells divide they might experience pressure not only from the cell layers above them, but also from neighboring cells within the forming primordium as was shown in the shoot apical meristem [18]. Therefore, the influence of KATANIN on AR formation was further studied here.

Of note, another recent study, in which the expression of tubulins and MAPs during AR induction in *Eucalyptus grandis* had found distinct expression patterns in easy to root juvenile cuttings compared to difficult to root mature cuttings [19].

In the current work we have asked how the expression of different MAPs, is regulated during AR formation and to what extent the loss of MT severing by the KATANIN ATPase influences these possible changes of expression. The expression of *MOR1* was not significantly different during AR formation in wild type and *bot1-1* plants, and the expression of GFP under its upstream sequence during early stages of AR primordium further confirms the importance of this MAP in AR induction.

## Materials and Methods

### Materials

All materials were purchased from Sigma (Rehovot Israel) unless otherwise mentioned.

### *Arabidopsis* plants, plasmids and transformation

*Arabidopsis* seeds were germinated and transformed as previously described [20]. To isolate the *MOR1* promoter, a fragment containing the upstream sequence of *MOR1* starting just before the ATG and including bp 14964640–14966828 from chromosome 2 was isolated by the primers described in S1 Table online. The 2kb fragment was ligated into the plasmid pBJ36 between NdeI and PstI, upstream to eGFP that was between BanHI and XbaI. Three independent transformants of *MOR1*_*pro*_:GFP were isolated. Other plants used were wild-type *Arabidopsis thaliana* ecotype Columbia or WS. KATANIN mutants *bot1-1* and *1–7* were from [21]. ARs were induced in cut etiolated hypocotyls that were incubated in MS supplemented with 1% sucrose and 10μM K-IBA in the dark.

### Microscopy

*MOR1*_prom_:GFP mean fluorescence was measured from the whole tissue imaged by the FL500 software from roots of 7d old seedlings which were grown on MS or on MS+10 μ M IBA for 16 hr (6d old seedlings which were transferred to IBA containing media over night). Three independent lines (*MOR1*_prom_:GFP 1, 6 and 7) were examined, from each 3 individual plants were measured for mean fluorescence, total of 9 plants). Mean fluorescence was also measured from hypocotyls which were excised from 7d old etiolated seedlings and from those excised similarly after 5d and incubate in the dark in MS +10 μ M IBA for 2d. For the closer examination of AR primordium, cut etiolated hypocotyls were incubated in MS media supplemented with 1% sucrose and 10 μ M K-IBA. Image acquisition was performed from hypocotyls of the line *MOR1*_prom_:GFP-7, after 1, 2, and 3d of induction. An Olympus (Hamburg Germany) IX81/FV500 laser-scanning microscope was used to observe fluorescently labeled cells with the following filter sets: for eGFP, 488-nm excitation, and BA505-525 were used; The objectives used were PlanApo UplanApo 10X 0.4 N.A, UplanApo 20X 0.7 N.A.

### Gene expression and clustering

Etiolated hypocotyls were excised from 7d old seedlings and incubated in MS + 1% sucrose with or without 10 μ M IBA. Total RNA was extracted from 3–4 biological replications at day 0 (the day of excision) and after 6h, 1, 2, and 3 d. Expression analysis of selected MAPs was performed by Nanostring (www.Nanostring.com, Seattle, Washington, USA). The primers used for hybridization are listed in S1 Table online. Total RNA was prepared using the plant/fungi total RNA purification kit of NORGEN BIOTEK CORP Canada according to manufacturer instructions and about 100 ng of each sample were used for the expression analysis. Expression of the different MAPs was normalized to the mean expression of two housekeeping genes IDH (isocitrate dehydrogenase At4g35650) and Alcohol oxidase (At1g03990).

To cluster genes that are similar by their expression profile, we scaled the gene-expression signals by subtracting their mean of the log_2_ signals over all conditions. This scaling removed the effect of expression level on the euclidean distance method. Two way clustering was performed by applying agglomerative hierarchical algorithm based on “ward” method on both the genes and the samples. The clustering results were plotted as two dendrograms attached to a “heatmap” using “Heatplus” R-package.

### Real-time PCR

RNA was extracted as above. RNA thus extracted was treated with DNase (Thermo Fisher) 1U/μg RNA at 37°C for 30 min followed by addition of 5 mM EDTA (Final concentration) and heat inactivation at 75°C for 10 min. Removal of genomic DNA was confirmed by carrying out PCR amplification of the following housekeeping (HK) genes, the expression of which was found constant under our experimental conditions; *Arabidopsis* Alcohol oxidase (AT1G03990) or tubulin β4 using (AT5G44340) using DNase treated RNA as template. No PCR product amplification in RNA samples was used as a quality control to ascertain the complete removal of genomic DNA. Quality checked RNA (1 μg) was used for cDNA synthesis using Maxima First strand cDNA synthesis kit as per manufacturer instructions (Thermo Scientific, USA). The amount of 2.5 μl cDNA was used as a template to carry out real time PCR analysis (Rotor gene Q, Qiagen) and quantify *MOR1* expression levels relative to HK genes. Primer sequences are listed in S1 Table. Thermo scientific absolute Blue qpCR SYBR green ROX mix (Thermo scientific, USA) and 1 μl of gene specific primer (10 μM) each were used as per manufacturer instructions. Real time PCR conditions were 95°C for 15 min, 40 cycles of 95°C for 10 s. 60°C for 15 s and 72°C for 20 s. Melting curve analysis (60°C–99°C) was also carried out to confirm the specificity of amplification. All the reactions were carried out in triplicate (both biological and technical) and an average Ct value was calculated. Expression was numerated as 2^δ Ct^ relative to HK as previously described [22].

## Results

### Adventitious root formation is hampered in plants carrying a mutation in *KATANIN*

**Fig 1:**
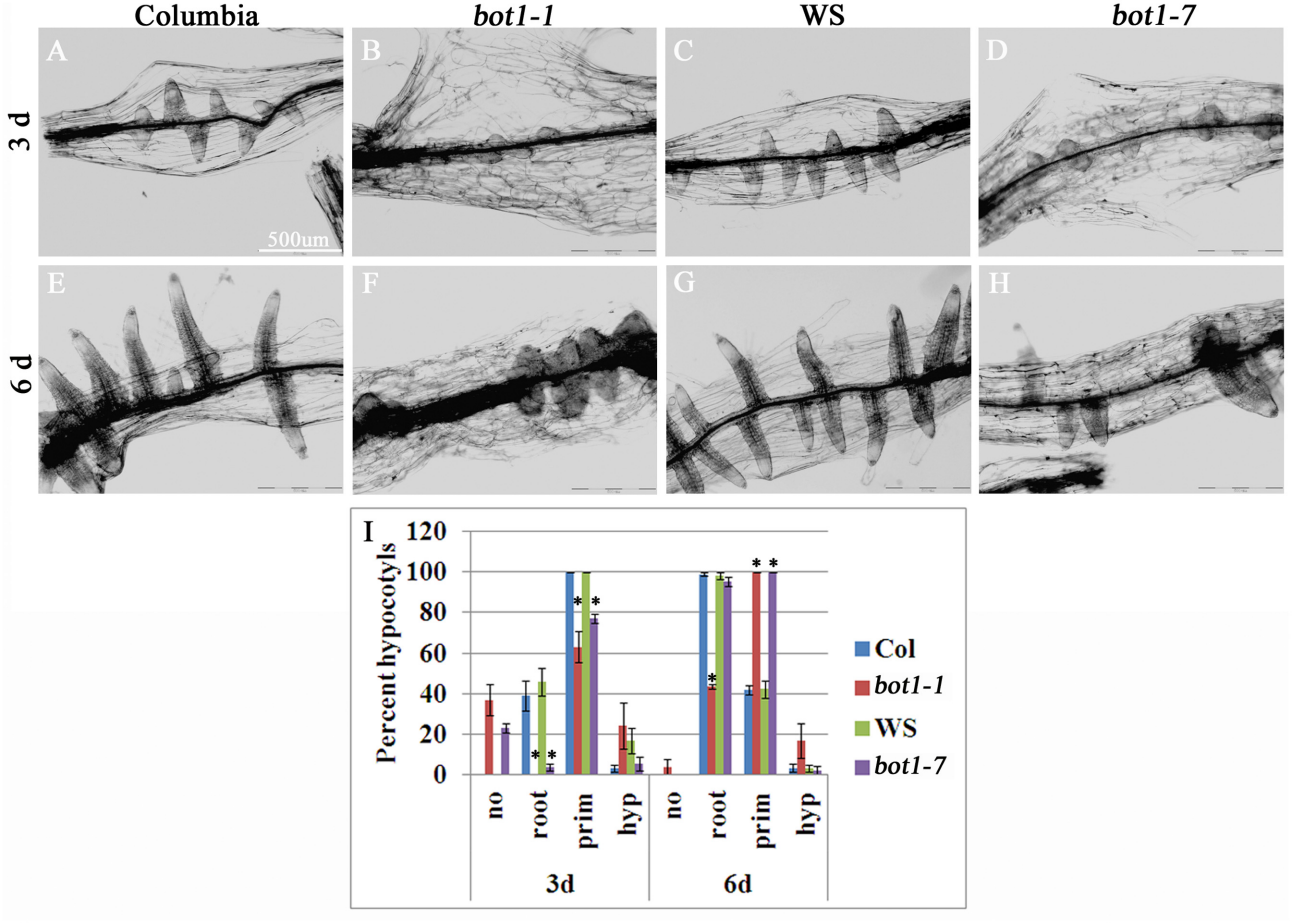
AR induction is hampered in *KATANIN* mutant plants. Hypocotyls (5-6mm in length) were excised from etiolated seedlings and incubated in MS with 1% sucrose and 10 μ M IBA for 3 days or 6 days. A-H representative images, scale bar = 500 μ m. I. Quantitative analysis of hypocotyls without roots (no), with emerged roots (root) or with AR primordium (prim). Asterisks show significant difference from control wild-type plants as determined by Scheffe analysis p<0.05. Scale Bar = 500 μ m.

Recently we showed that *bot1-1* mutant seedlings are hampered in AR formation [8]. Here we examined to what extent the reduction of *KATANIN* expression affects AR formation in cut etiolated hypocotyls treated with IBA. Two *KATANIN* mutants were used: *bot1-1*, which is in the Columbia ecotype background, and *bot1-7*, which is in the Wassilewskija ecotype (WS). Fig 1 shows that while 100% of wild-type hypocotyls had developed root primordium 3 days after induction, only 63 and 77% of *bot1-1* and *bot1-7* hypocotyls had detectable root primordium at that time respectively. In addition, while about 40% of wild-type hypocotyls showed emerged roots, 0% of *bot1-1* and 4% of *bot1-7* had such roots 3 days after induction. To determine whether the reduced KATANIN expression affected root induction or root emergence, hypocotyls were also examined after 6d. It was found that while 100% of hypocotyls of wild type had roots after 6d, more than 90% of *bot1-7* and 40% of *bot1-1* had roots at that time, indicating that once formed, root primordium eventually emerged in the mutant plants (Fig 1). Taken together, efficient AR induction by ectopic application of IBA requires KATANIN.

### Comparative MAP expression during AR formation in *bot1-1* and wild-type plants

**Fig 2:**
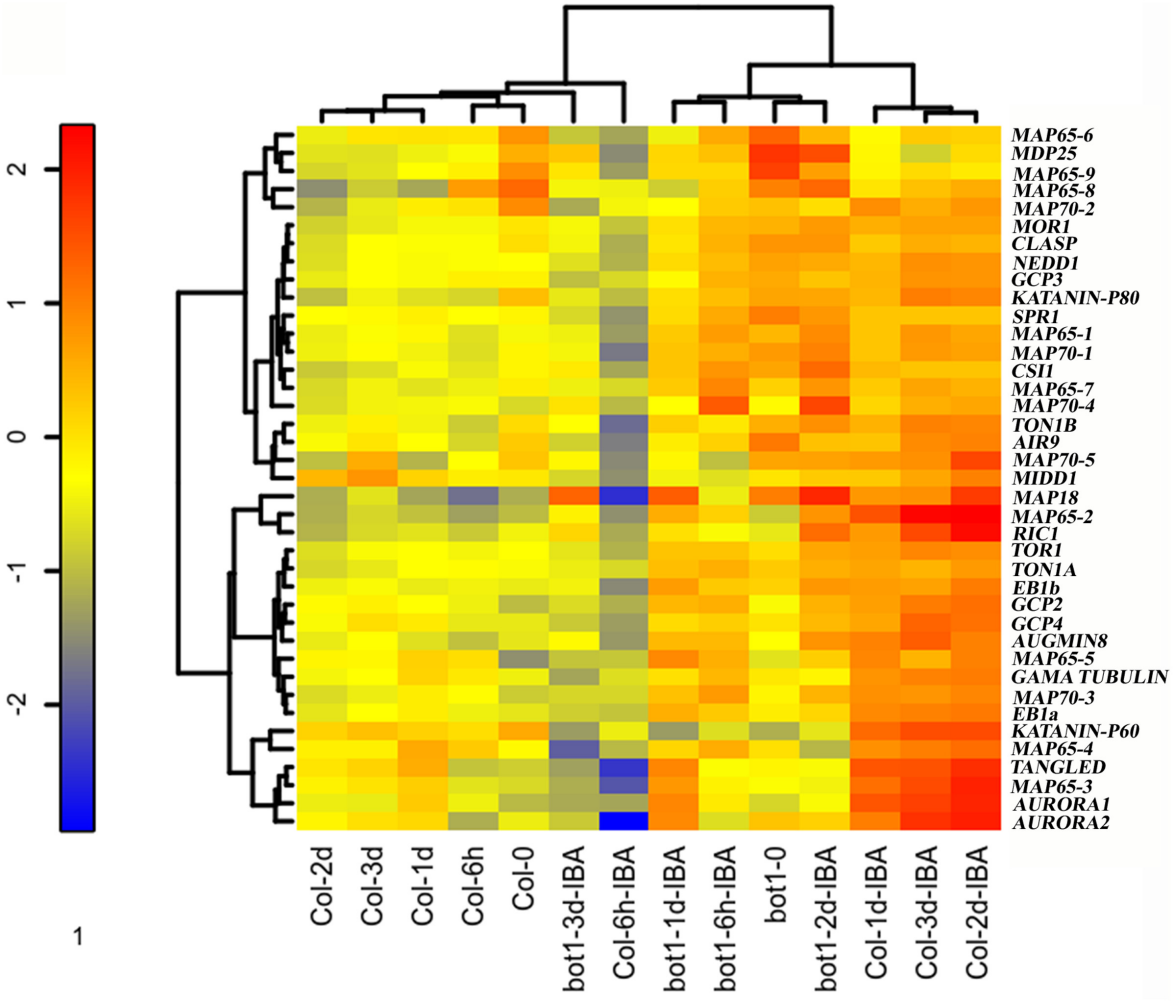
The kinetics of expression of various MAP-encoding genes in wild-type and *bot1-1* plants during AR induction. RNA was extracted from 3–4 biological replicas at the indicated time points and expression analysis was performed by the nanostring method. Scaling of gene expression signals was performed by subtracting the mean of the log_2_ signals. An agglomerative hierarchical algorithm was applied with the “ward” method parameter on both the genes and the samples and a “heatmap” was illustrated by “Heatplus” R-package.

**Fig 3:**
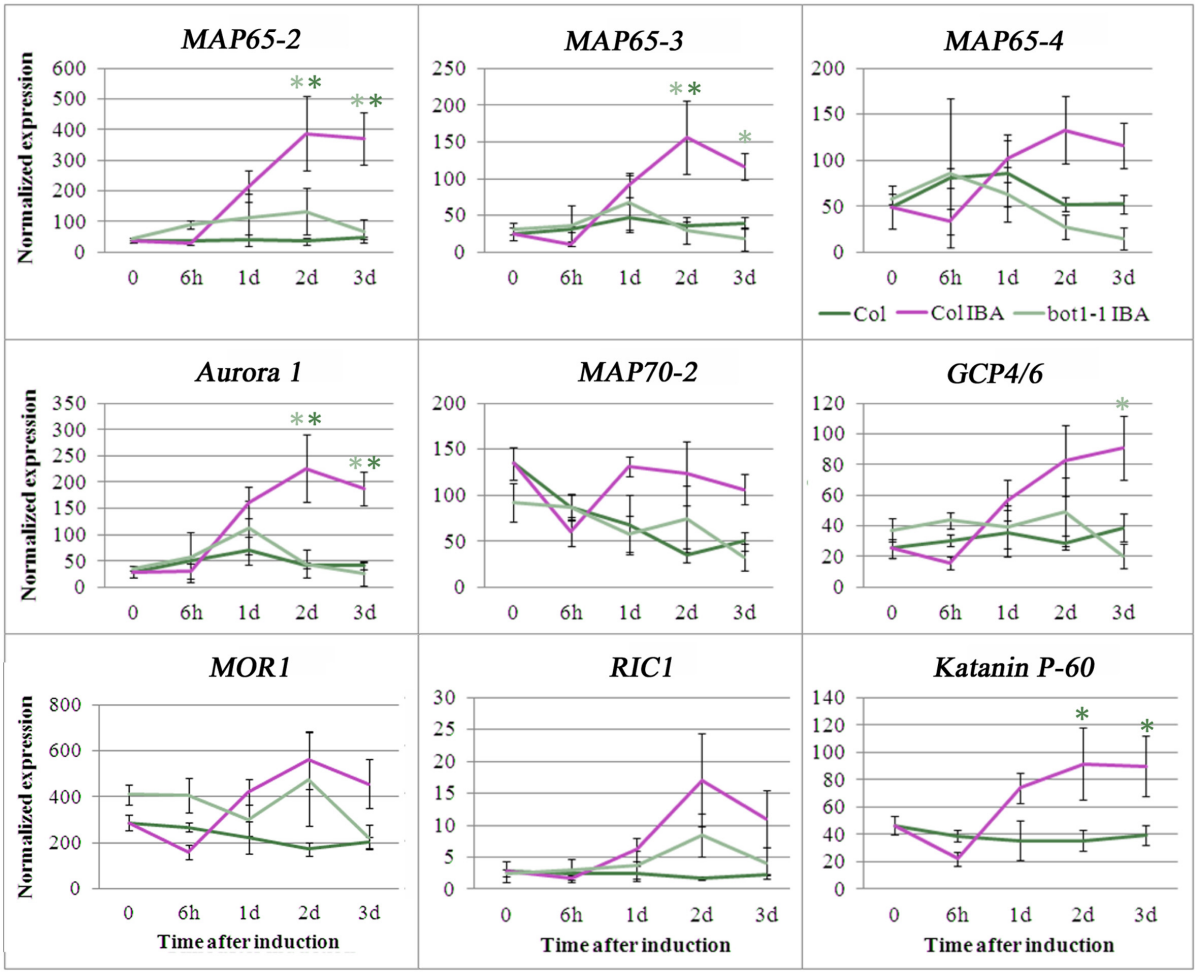
Statistical analysis of the expression of several MAPs during AR induction in wild-type and *bot1-1* plants. Hypocotyls (5-6mm in length) were excised from etiolated seedlings and incubated in MS with 1% sucrose and 10 μ M IBA. RNA was extracted at the indicated time periods and expression of the various transcripts was analyzed in 3–4 biological replicates by the nanostring method. Light and dark green asterisks show statistically significant differences of expression in Col+IBA in relation to *bot1-1*+IBA or Col respectively, as determined by Scheffe analysis p<0.05. Col = wild type *A*. *thaliana* Columbia.

In continuation to the above and our previous study [8] we asked whether the expression of *MOR1* or other MAPs is changed during AR induction and whether there is a difference between the wild-type and *bot1-1* plants in which AR induction is hampered. The expression of 39 different MAPs was determined in etiolated wild-type or *bot1-1* hypocotyls, before and after they were induced by IBA to form ARs. RNA was extracted at 0, 6h, 1d, 2d and 3d after excision to cover all stages of AR formation as previously determined [8], and expression analysis was performed using the nanostring method [23]. Expression of MAPs was normalized and cluster analysis of the results was performed to determine relationships in expression. Three main groups were distinguished (Fig 2): one group included the hypocotyls from wild-type plants that were not treated with IBA and that were harvested 0, 6h, 1d, 2d and 3d after induction, which were all clustered together. These samples did not develop AR primordium and were most distantly positioned by the cluster analysis with respect to the group of hypocotyls that were treated with IBA and did develop AR primordium. In between these two groups was that of hypocotyls from *bot1-1* that were treated with IBA and had fewer AR primordium. Interestingly, 6h after IBA application there was a strong reduction of MAP expression in hypocotyls of wild-type plants. There was a mild reduction in MAP expression in the hypocotyls not treated with IBA, but in *bot1-1* hypocotyls treated with IBA no such reduction was observed 6h post induction. Later on a gradual increase in MAP expression was observed until day 3 in wild-type hypocotyls treated with IBA. In contrast, no such major increase was observed in the wild-type hypocotyls not treated with IBA. In *bot1-1* hypocotyls treated with IBA, there was an increase in MAP expression until day 2 and then a decrease on day 3. An examination of the expression of *MOR1* showed upregulation 2 days after induction both in the wild-type and *bot1-1* plants. Similarly, the expression of *MAP18*, for example, was induced along with ARs both in wild type and *bot1-1*. In contrast, while the expression of *MAP65-2 MAP65-3*, *AURORA1*, *AURORA2*, *and TANGLED* was induced in wild-type plants but not in *bot1-1* plants, the expression of *MAP65-8* and *MDP25* was reduced along AR induction in wild-type plants but not in *bot1-1*. Statistical analysis of the expression levels of, for example, *MAP65-2*, *AURORA1*, and *KATANIN p60* (Fig 3) revealed significant differences between control and IBA-treated samples in wild type plants. Taken together, these findings suggest that a MAP expression pattern change takes place during AR induction in *Arabidopsis*. The differences between wild type and *bot1-1* in this respect, suggest that AR induction and MAPs expression changes are KATANIN dependent. Nevertheless, it remains unclear whether the difference in MAP gene expression is the consequence or the cause of the inhibition of AR induction in *bot1-1* plants.

### Expression of GFP under the *MOR1* promoter increases during AR induction and accumulates in the pericycle and AR primordium

**Fig 4:**
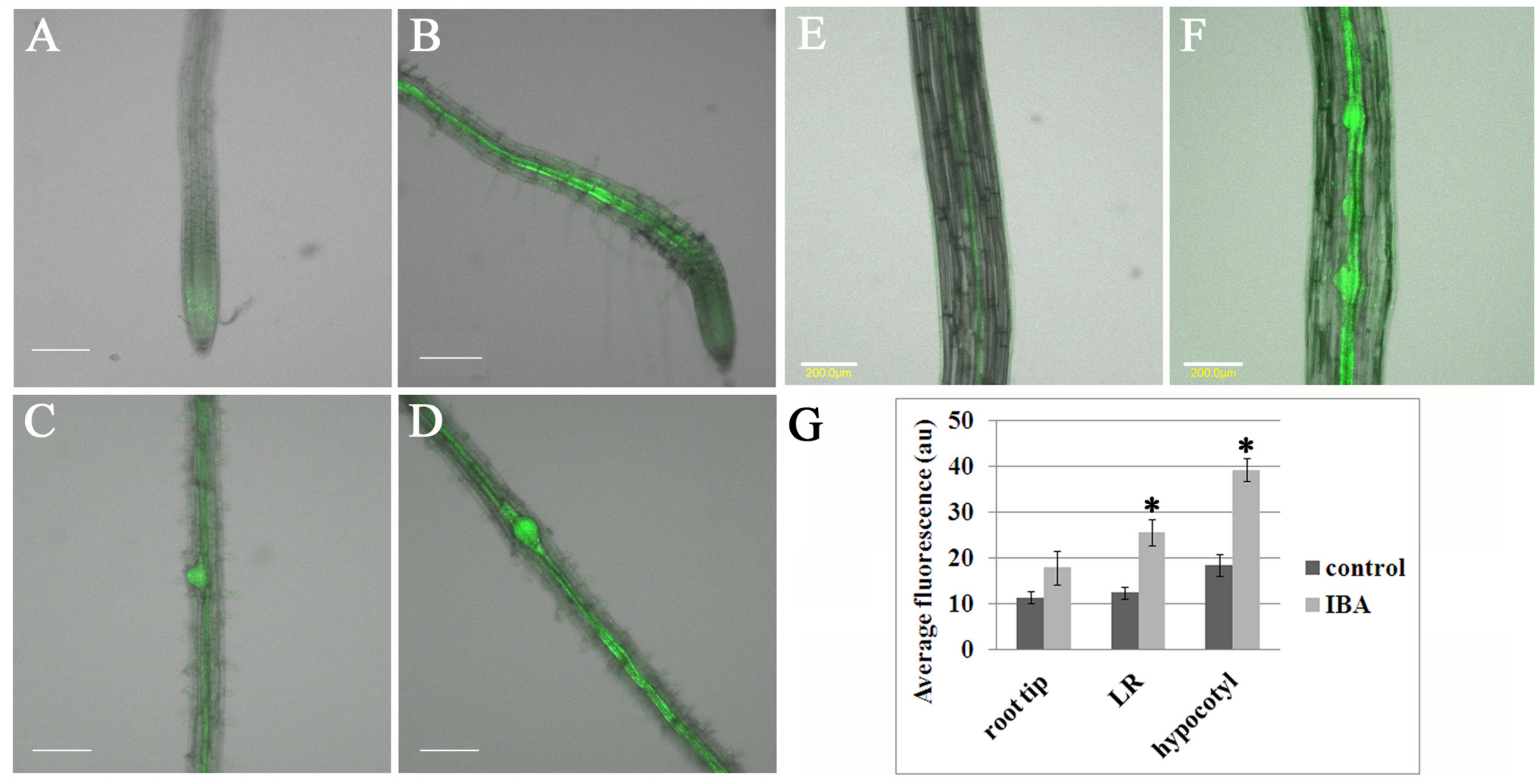
GFP expressed under the *MOR1* promoter is upregulated in response to IBA in the root and accumulates in LR and AR primordium. (A-D) *MOR1*_*pro*_:*GFP* seedlings were grown on MS medium for 6 days. Half were then transferred to plates containing MS and 10 μ M IBA for 16h, after which a comparative examination by confocal microscopy was performed. (E-F) In another experiment, etiolated hypocotyls were excised (E) or excised and incubated in MS and 10 μ M IBA for 2d (F). (G) Fluorescence was measured in 3 plants from three independent transgenic lines (total of 9), asterisks show statistically significant differences as measured by Scheffe analysis p<0.05. Scale bars in A-F are 200 μ m.

Our analysis indicated that *MOR1* is upregulated during AR induction and that this is not altered by the *bot1-1* mutation. To elucidate this, we documented *MOR1* promoter activity during AR induction. The *MOR1* upstream DNA sequence was cloned in fusion with the gene encoding GFP. In addition, *MOR1* mRNA levels were determined by real time PCR in roots and hypocotyls induced to form AR primordium in wild-type and *bot1-1* plants. First, the intensity of GFP fluorescence was compared in roots of intact plants that were grown on MS with those that were grown on MS but transferred to MS+ IBA for 16 hrs. Fig 4 shows accumulation of GFP in root tips and lateral root (LR) primordium of plants at similar age, either transferred or not to IBA 16h before examination. GFP average fluorescence intensity analysis indicates that significantly more GFP accumulates after IBA treatment in root tips and LR primordium. Higher levels of *MOR1* mRNA were detected by real time PCR in the roots transferred to IBA, albeit the difference was less significant (Scheffe analysis p = 0.09) (S1 Fig). When cut etiolated hypocotyls were incubated in MS supplemented with IBA, it was found that GFP expression under the *MOR1* promoter was greatest in the pericycle and in AR primordium (Fig 4). *MOR1* mRNA levels were higher in etiolated hypocotyls induced to form AR primordium by IBA, albeit, with a lower significant difference (Scheffe analysis p = 0.2) (S1 Fig). This might be the result of the heterogeneous tissues used for RNA preparation, which included transcripts from non-responsive cells. Validation of *MOR1* expression by real time PCR confirmed no significant differences between wild type and *bot1-1*. In both plants *MOR1* expression increased 2 days after AR induction by IBA (not shown), which is in agreement with the nanostring data.

**Fig 5:**
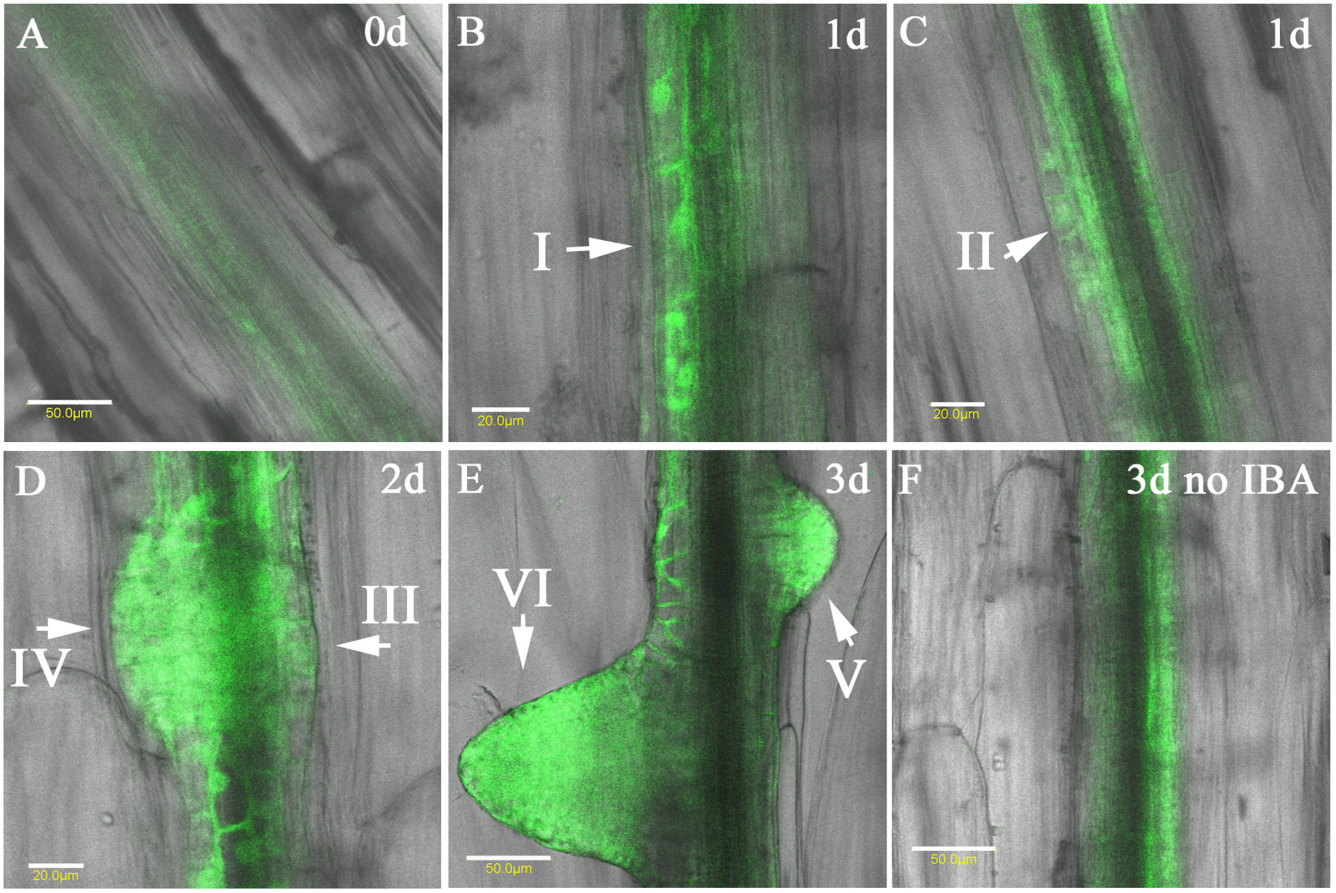
Expression of GFP under the *MOR1*_prom_ during AR induction. Etiolated hypocotyls were cut and induced to form AR by 10 μ M IBA. GFP fluorescence was followed before induction (A), and 1, 2, and 3 days post induction (B—E). (G). A hypocotyl that was incubated in MS without IBA for 3 days. The different primordium stages as in [8] are shown by arrows. Scale bars in A, E, F are 50 μ M and in B -D are 20 μ M.

To follow the expression of GFP under the *MOR1*_prom_ in initial steps of AR induction we have induced AR in etiolated hypocotyls and imaged them after 1, 2 and 3 days. Fig 5 shows that before induction, a faint GFP signal was observed in the pericycle, thereafter GFP was detected in all stages of AR formation as previously defined [8]. After 1d GFP was observed in the founder cells (stage I, Fig 5B) and in the first layer formed by periclinal cell division (stage II, Fig 5C). After 2 and 3 days accumulation of GFP in AR at stages III-VI was observed (Fig 5D and 5E). When no IBA was applied, GFP fluorescence was increased in the pericycle compared to samples on day 0, but no ARs were formed (compare Fig 5A to Fig 5G, measurements as in Fig 4).

## Discussion

In this study we have determined that the expression of MAPs is regulated during AR formation and that this regulation is perturbed when the MT severing protein KATANIN is reduced. Thus, while KATANIN can directly influence MTs organization patterns [12, 24] it might also indirectly affect the expression of other MAP-encoding genes. This is in agreement with a recent report showing that disruption of MTs by oryzalin led to the alteration of the expression of auxin-regulated genes [25].

MTs are sensitive to mechanical signals [26–29] and are responsive to tissue level stress patterns [13, 29–31] a response that possibly requires KATANIN-dependent MT severing [13]. Therefore, it is conceivable that in AR primordium cells, MTs are sensitive to the pressure exerted by the upper cell layers, and that KATANIN is required for MTs perception of these mechanical signals. It is hypothesized that this mechanical perception is part of the ARs differentiation program. Disruption of MTs was shown to affect the polar localization of PIN1 in the shoot apical meristem [32] and in AR primordium [8]. In addition, RIC1 that promote MTs bundling [33] and activates KATANIN [9], is an effector of the GTPase ROP6 that is activated by auxin [34]. The three latter genes were confirmed to participate in the signaling from auxin to MTs [11]. These suggest a complex cross talk between MTs and auxin that might be perturbed in *bot1-1* plants, which could lead to loss of coherent auxin transport within AR primordial cells, inappropriate regulation of various MAPs expression by auxin and to inhibition of AR formation.

Taken together with our previous findings that loss of *MOR1* function inhibited AR formation and over expression of the RIC1 increased AR formation [8], the current study results confirm an important role for MT remodeling by MAPs during AR induction.

Given that MTs are involved in cell division [35, 36] and elongation [37–39] this is not surprising, yet our data also suggest a role for MTs in coordinating dividing cells to form a functional organ. Initial indications for this additional role were shown when AR induction in the presence of subtle perturbation of MTs resulted in amorphous clusters of cells instead of roots [8] showing that cell division continued but coordinated differentiation did not. The findings here further support this proposed function.

Expression profiles of MAPs in wild-type plants treated with IBA for 0–3 days, during which time AR formed, were compared to expression profiles of untreated samples in which no AR were formed and to *bot1-1* samples treated with IBA in which fewer AR were formed. Interestingly, all the samples within each of the above groups clustered together, indicating that MAPs expression remodeling during AR induction was influenced by the IBA treatment and the *bot1-1* mutation. Down regulation of some MAPs was observed in wild-type plants 6h after IBA application, which is interesting and yet to be explained. Of note, the expression of MAPs that are known to function during cell division such as *MAP65-4*, *MAP65-3*, *AURORA1*, *AURORA2* and *TANGLED*, [40–42], increased in the IBA-treated wild-type plants but not in *bot1-1* plants. Interestingly, the expression of *KATANIN* itself increased in wild-type plants induced by IBA but not in IBA untreated plants, indicating its importance. The expression of MAP65-8 and MDP25 [43] decreased only in the wild-type plants but not in the *bot1-1* mutant after IBA. MAP65 family members affect MT arrays in different ways. It has been found, for example that while MAP65-1 can promote MT polymerization, MAP65-6 does not [44]. In addition, while MT bundles formed in the presence of MAP65-1 were more resistant to cold, those formed in the presence of MAP65-6 were more resistant to salt [44]. Different MAP65s were shown to affect differently the flexibility of MTs *in vitro* [45]. The flexibility of MTs is important to allow bending deformation for co alignment [46]. In addition, MTs that wind around the box-like geometry of plant cells, deform to adjust to the physical constraints [47, 48]. In this respect it is worth noting that CLASP, a microtubule-associated protein [49–51], accumulates at sharp cell edges and facilitates the successful growth of MTs around these edges [47]. *clasp* plants exhibit auxin-related phenotypes, including abundant lateral roots [49, 50] and the formation of callus on etiolated hypocotyls [52], suggesting its involvement in cell proliferation and lateral organ differentiation. Taken together, it is concluded that differential expression of MAPs affects MT nucleation, polymerization, dynamics, ordering, bundling, flexibility and stability, and that the fine-tuning of MTs behavior is important for coordinated organ differentiation. The distinct MAP gene expression profiles in *bot1-1* and wild-type plants suggest that MAP expression changes during AR induction by IBA is MT-organization dependent. It should, however, be taken into consideration that RNA was extracted from the whole hypocotyl and not preferentially from primordium, so it cannot be ruled out that some MAP transcripts are regulated differently within the dividing cells.

For this reason the activity of *MOR1* promoter has been determined in cells of AR primordium. It was found that the promoter of *MOR1* is induced in the primary root tips and LR, in the pericycle of the hypocotyls and in AR primordium following IBA treatment of 16-72h. This suggests that the expression of *MOR1* is a target of transcription factors that in turn are direct or indirect targets of auxin. Curiously, GFP under the *MOR1* promoter accumulated, albeit to lower levels, in the pericycle of hypocotyls after 2-3d of incubation in liquid MS even when no IBA treatment was applied and no AR primordium were formed (Fig 5E). This suggests promoter responsiveness to other signals, such as wounding. In addition, while GFP under the *MOR1* promoter is upregulated at all stages of AR induction, it is also found in all pericycle cells, and not preferentially in those that give rise to AR primordium, suggesting that it is necessary but not sufficient for AR induction.

Taken together it is concluded that precise alterations of MAPs expression occurs during AR induction by IBA. In the background of a mutation in *KATANIN* AR induction is inhibited and some MAPs expression is altered, suggesting relationships between AR differentiation and MT remodeling that depends on *KATANIN*. In addition, GFP under *MOR1* regulatory sequence is specifically upregulated in the pericycle and AR primordium.

## Supporting Information

S1 Fig Expression of *MOR1* mRNA after IBA induction as determined by real time PCR. (TIF)

S1 Table The primers used in this study. (DOCX)
