# Supplementary figures and images for: Analysis of Microtubule-Associated-Proteins during IBA-Mediated Adventitious Root Induction Reveals KATANIN Dependent and Independent Alterations of Expression Patterns
Source: PLoS One. 2015 Dec 2;10(12):e0143828. doi: 10.1371/journal.pone.0143828 (PMC4668071; doi:10.1371/journal.pone.0143828)

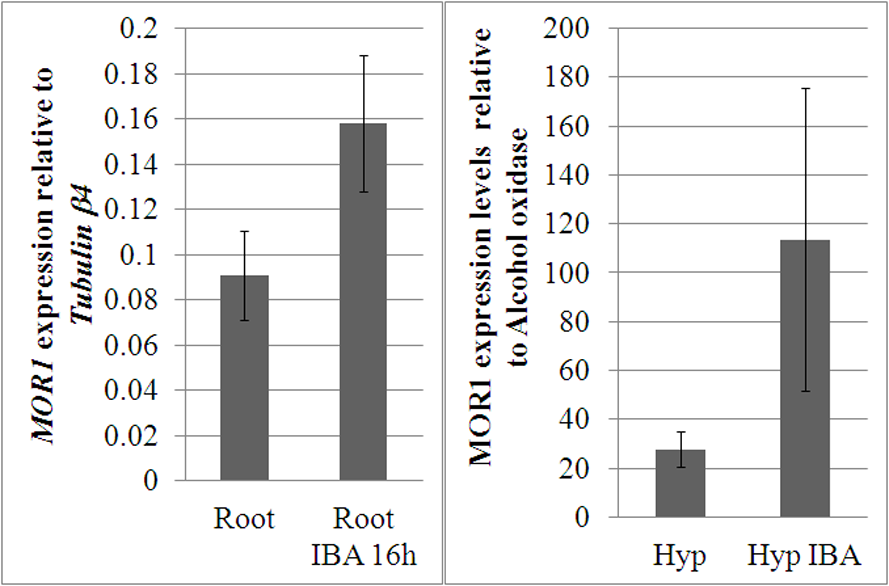

Supplement: S1 Fig — (TIF) [file pone.0143828.s001.tif]
