# Supplementary material for: Analysis of Microtubule-Associated-Proteins during IBA-Mediated Adventitious Root Induction Reveals KATANIN Dependent and Independent Alterations of Expression Patterns
Source: PLoS One. 2015 Dec 2;10(12):e0143828. doi: 10.1371/journal.pone.0143828 (PMC4668071; doi:10.1371/journal.pone.0143828)
Supplement: S1 Table — (DOCX) [file pone.0143828.s002.docx]

Table 1a primers used for real time PCR

| Primer name | Primer sequence | Target gene | accession No. |
| --- | --- | --- | --- |
| TUB4 F | 5’ CTGTTTCCGTACCCTCAAGC 3’ | Tubulin 4 | [At5g44340](http://www.arabidopsis.org/servlets/TairObject?type=locus&name=AT5G44340) |
| TUB4 R | 5’ AGGGAAACGAAGACAGCAAG 3’ | Tubulin 4 |  |
| MOR1 N-TERM F | 5’ CGACGGAGGATGAGAAGTTATT 3’ | Microtubule organization 1 protein | At2g35630 |
| MOR1 N-TERM R | 5’ ATCGACGTTAGCTTCGTTCC 3’ | Microtubule organization 1 protein |  |
| AT alc oxidase F | 5’ ATGCAAGTGTTCTGCCTACA 3’ | Alcohol oxidase | At1g03990 |
| AT alc oxidase R | 5**’**TCTTCTTCATTAACTCGGCTATTCT 3’ | Alcohol oxidase |  |

Table 1b. The primers used to clone the mor1 promoter

| Primer name | Primer sequence | purpose |
| --- | --- | --- |
| mor1 promoter FW | TTATGGCACCGTTCAAGCATAATATAGCTA | purification |
| Mor1 promoter R | TACTCCACGAAATTATCTCTCCGACGGTAT |  |
| Promoter NdeI | GGAATTCCATATG TTATGGCACCGTTCAAGCATAATATAGCTA | Cloning into Bj36-GFP |
| Promoter PstI | AACTGCAG TACTCCACGAAATTATCTCTCCGACGGTAT |  |

Table 1c. The primers used for NanoString analysis

| Name | Accession | Target Region | Target Sequence |
| --- | --- | --- | --- |
| AIR9 | NM_129022.6 | 3806-3905 | AGCAAATCCTATGGGGTTGGAGCTTTTAATTCCTGACTGCTGTGAGAAACAAGAGGTTGTGCCTCATAAAACTTATTTTGGGGGCCATGAAGGTGTTGGA |
| Alc oxidase | NM_100280.2 | 250-349 | GTGGCAGAGCTACTGGCGACCAAGGCAATACCATTGACAGTTTTAGTGGTGAGAATAGTTTTGAGAATACTCACATTCAGACTGGGGACGTTACTGCTTT |
| AtMAP65-7 | NM_101338.3 | 969-1068 | CGAGCTTACATAGGACTGACCATGAGCAGTCTACAAACATTTCTGATGATACATTGGATGGTCTACACCACATGATTCATAAGCTGAAAACCGAAAGGAG |
| AtMAP65-8 | NM_102557.1 | 263-362 | GAAAAGTTGACCATGCCAATGTTTCGAGGTCTCGTCTACATCAAGAGCTGGCAGAATCTGAAGCCGAACTCACCCATTTTCTTTTGTGCCTCGGTGAAAG |
| CSI1 | NM_127781.4 | 3962-4061 | CAAGAGGAAGCTGCAACAGGTCTTTTAGGCATCTTATTTAGCAGTGCTGAAATTCGAAGGCATGAGTCAGCATTTGGCGCGGTCAGCCAACTTGTAGCTG |
| AtMAP65-5 | NM_129429.2 | 1430-1529 | GAGGGGTCCCCTTCTTATGCGATAAGCAACCGTTATTACAAACACTAGAAGATGACATTGTTATCCGTGCTCAAAGAGAAGAGGAAAAGCGTCAATTCCG |
| MIDD1 | NM_202695.1 | 630-729 | ACACGGAATGGATTCTACCGCTCTGTCTTCTGCCATTAACGAAGTTCAGAAACTGAAATCGAAGCTCTTTGAGTCCGAGTCTGAGTTAGAGCAGTCTAAG |
| GCP4/6 | NM_115236.3 | 2333-2432 | ATTGTAAAATGCCAGACAGTCCTGGTGGAAGAATCTCGTTCCGAAGCTTTTTCAGACATGAGCTGATCCGAAGGTTTAATGCAACAACTATGACACGAGA |
| AtMAP65-4 | NM_115948.1 | 66-165 | TTATAAGCGAAAGGTCGAGGAGGCTAGTCGGGGTAAAGCGAATTTGCTGAAAGAAATCGCTGTTGGCAGAGCAGAAATTGCAGCTATTGGCTCTTCTATG |
| AtMAP65-2 | NM_118810.3 | 1168-1267 | ATGCTCGTGCTCACATAGAAATAAAACCAGAGGTTGTTCGTGAGAGGATCATGTCGTTGATTGATGCTGGGAATACTGAACCTACTGAGTTACTGGCTGA |
| AUGMIN8 | NM_119217.2 | 640-739 | TTCGTTAGCTTGAATGGATGTAGCAACAGATACTACAAGGCGGCGTCTTCTTCCATCCGACAAGAACAATGCAGTTGTTGCCACCCGCCGTCCTCGAACA |
| Isocitrate dehydrogenase | NM_119730.2 | 443-542 | TGTCAATTGCATCAATGTCCCTGGATTAGTGACGCGACACGAAAATGTTGATATCGTTGTGATAAGAGAGAACACTGAAGGAGAGTACTCAGGTCTCGAG |
| katanin p80 | NM_122250.2 | 3169-3268 | GTCTGCATTGTTCTAATTTTGGTGTGTAGTAGAGAGGGACAGAGAGAAAGAGAGAGAGCCATTGGGCTGTGTATTGTTCTTATGAGAGTACTGCTTGAGT |
| MAP18 | NM_123828.3 | 202-301 | AGGAGGTCGTCGTGAAAACCGAAGAACCGGCCAAGGAAGGAGAAACTAAACCGGAGGAAATAATTGCAACCGGCGAGAAAGAGATAGAAATAGTTGAAGA |
| AtMAP65-9 | NM_125619.1 | 1031-1130 | CGGTAGAGAATGTAATCAAAGCCATTGAATCAGGAGATGTGAACCCTGAAAATATACTAGAACAGATCGAGTATCGAGCTGGGAAAGTGAAAGAGGAAGC |
| Aurora1 | NM_119436.2 | 190-289 | GCGATTTCGACATCGGTAAGCCTCTTGGCAGAGGCAAATTCGGTCACGTCTATCTCGCCAGAGAAAAACGGAGCAATCACGTTGTCGCTCTAAAGGTTCT |
| Aurora2 | NM_128148.3 | 803-902 | TCCCAAACCCATTGTGTCATCATCTGCAAAGGACCTGATTAGCCAGATGTTGGTTAAGGAGTCTACGCAACGTTTGGCCTTGCACAAGCTTCTTGAACAC |
| EB1a | NM_114637.4 | 696-795 | ATTCCAAAGTCACTGCAAACAAACAATAACCATCCTCCACCCAATTCCAGTTCAGTTGGTCTTAGCAAAGCATCAGGGCCCAAGTCAGCAAAAGCAGCTG |
| EB1b | NM_125644.2 | 1145-1244 | CAGCTGCTGCAGAGACCCAAACTTAAAGTTGTGACAAATAAAAGTAGAGGGCAACAATGATCCATTGCCTTGTCTTAGAAGATAGAATCACTTGGAGAGG |
| AtMAP65-1 | NM_124905.4 | 547-646 | TATGTGGAGATATTGCTGGAGGTTTGAGCAATGAGGTTCCTATAGTCGATGAGTCTGATTTGTCACTGAAGAAATTAGACGATTTCCAGAGCCAACTCCA |
| AtMAP65-6 | NM_126252.3 | 2038-2137 | GGAAGATACTGTTTCTACTACCTACACATCGATTTATAGCTCTGAGCCAGACTCGCCTCTCCAAGGCTGACTTGACTCTCTTTTCCACCGGATTTGTGGA |
| MAP70-1 | NM_105476.4 | 245-344 | CGGAAGGAATTTAGATCGGAGTAGTCGGAGATGTCTGACGTTTCGGCTGACGGTGGTTTTTTGTCGGCGGAGCAAGCTACAACGCCTGTTGCGATTCCGA |
| MAP70-2 | NM_102311.6 | 2191-2290 | GAGAGTTTGTGACATAGATTTGGTGTTAAAGTGGTTAGAGGGAGGAGAAGAAAAGCCGAGGGAGAGAGTGAGAGATGTTGACATTTTGGCTAACATTTTC |
| MAP70-3 | NM_126236.2 | 537-636 | AGCTATTCTCGCTCCTTTAGAGGCTGAACTCAAGTTGGCGAGACATGAAATTGTTAAACTTCAAGATGACAACAGAGCATTGGACCGTCTAACCAAGTCG |
| MAP70-4 | NM_101353.1 | 1005-1104 | TGCGAAGGTAAATGCAAACAGAGTTGCTACTGTTGTAGCTAACGAATGGAAAGATTCTAATGACAAAGTGATGCCTGTAAGGCAGTGGCTTGAAGAACGT |
| MAP70-5 | NM_117827.2 | 512-611 | GAAGCAGAGAGAATCTTGAGAAGTGCCCTTGAACGTGCTTTGATTGTTGAAGAGGTCCAGAACCATAACTTTGAACTTCGACGACAGATTGAGATATGCC |
| tangled | NM_111406.4 | 1181-1280 | AATCAAATCTCCTCCCACATCAGCATCTAAGTTTCAGGTCAAGATCAGGAGCCCACCTAAGGTCTTGGTTTCTCCTACAAGAAATGGCAGTAACTCGGTT |
| katnin p60 | NM_106684.4 | 323-422 | CGATCCTTTGGCCCGGACCAAATGGATGAACGTTAAAAAGGCCATAATGGAAGAGACGGAAGTTGTGAAGCAATTGGATGCAGAGAGGAGAGCATTTAAA |
| MOR1 | NM_129117.4 | 3854-3953 | TTCTTCCAGAACTTTTCAACACATTGAGGGATGAGGAATACTGCATGACGGAAGCAGAAGCTGCAATATTTCTGCCTTGTTTAGCGGAGAAGTTGGGGCA |
| NEDD1/GCP-WD | NM_120679.3 | 1907-2006 | GATCAGAGACTTCTCTTCAACCTTTGAGACATCCTCAACACAGACAGACAACAACTTGCCTTCAAGCCCATTGTTTACAAAAGGCATCACAGCTCCTGGT |
| ton1A | NM_115357.3 | 735-834 | AACTGGAGACAAGGTAACCAAGATACACATGAAGAGGTAACAAGAGCTTCAGCGGCGCTGGAAAATCTACAGCTTGATAGGAAAACTCGGAACTTAACAT |
| ton1B | NM_115358.4 | 729-828 | GACGGTCGGTTTCTGCATCTCAAGCATCGGGAGCCGCTACCTCAGGGTACAGAAAAGACGAGAGCAATTGGAGATATGACACTGAAGATATGCCGGAAGA |
| GCP3/SPC 98 | NM_120751.3 | 365-464 | CTCGCTACTCAGGGTAAGTCCTCCGACGCACTCGCTTTTGCCGATCTCTACACTAAGTTCGCTTCCAAAACTGGACCTGGCAGTGTCAATAACAAATGGG |
| AtMAP65-3 | NM_124539.3 | 1274-1373 | CCCACTTGTTGCCAGTGTCAGACAGTGCTATAGATCAGACTATCGTGGCGATAGAATCTGGTATTGTGGATGCTACAATGGTCCTGGAGCATCTTGAGCA |
| clasp | NM_179666.4 | 2218-2317 | TTCAAAGGACTCAGGAAGATCATCTTACCGTGGCAATCTGTTGTCCGAGTCTCATCCTACTTTTTCATCCTTGACCGCTAAACGGGGCTCAGAGAGAAAT |
| MDP25 | NM_179081.3 | 167-266 | AAAATTCAAGAAGTTATTCGAGAAAAATAGTGCTAAGAAGGCTGCTGCTGCTGAAGCTACCAAGACCTTTGATGAATCTAAGGAAACAATCAACAAGGAA |
| spr1 | NM_126416.2 | 109-208 | CAGCTGTGGTGGAGGTCAAAGCTCATTGGATTATCTCTTTGGTGGTGACGCTCCTGCTCCTAAGCCAGTTCCAGCTCCTCGTCCCGCTCCTACTGAGTCT |
| tor1 | NM_118840.4 | 510-609 | GAGATACGATTGGTGCTTTGTCTGGGATTTATCTCAAGGGAAAAGAAGAGGGGACTAATACTGGTTCAGCGTCGTTAGCGGTGGGTTTGTTCGTTAAGCC |
| gama tubulin | NM_116030.1 | 597-696 | TGATTGTGTTGTTGTCCTTGACAACACTGCTTTGGGAAGGATTGCTGTGGAGCGTCTACATCTGACAAATCCTACCTTTGCTCAAACGAATTCTCTAGTG |
| GCP2 | NM_120644.2 | 20-119 | TAATGAGTATAGTTTGATCATCCTCTTCAGGCGTAGTAGTCTCGAAACACTTAGGTAGGGTTTTACAGAGGAAGAAGATGCCGAGGGAGATAATTACGCT |
| RIC1 | NM_128906.2 | 507-606 | GCAGCTTCTCCAAATCATAACGGGTCTCCGCCTAGAAAAAGTAGCGGAAATGCTGCATCATCAGATGAACCATCAAAGCATTCAAGACATAACCGAAGCG |
